# Relationship of Preexisting Vertebral Fractures and Endplate Injury to Intervertebral Bridging Ossification After Balloon Kyphoplasty for Osteoporotic Vertebral Fractures

**DOI:** 10.1155/joos/1886674

**Published:** 2026-02-23

**Authors:** Toshiaki Maruyama, Naosuke Kamei, Toshio Nakamae, Yoshinori Fujimoto, Kiyotaka Yamada, Kazuto Nakao, Fadlyansyah Farid, Hiroki Fukui, Nobuo Adachi

**Affiliations:** ^1^ Department of Orthopaedic Surgery, JA Hiroshima General Hospital, Hatsukaichi, Japan, hirobyo.jp; ^2^ Department of Orthopaedic Surgery, Faculty of Medicine, University of Miyazaki, Miyazaki, Japan, miyazaki-u.ac.jp; ^3^ Department of Orthopaedic Surgery, Graduate School of Biomedical and Health Sciences, Hiroshima University, Hiroshima, Japan, hiroshima-u.ac.jp; ^4^ Department of Spine and Spinal Cord Surgery, Amano Rehabilitation Hospital, Hatsukaichi, Japan; ^5^ Department of Orthopaedic and Traumatology, Faculty of Medicine, Hasanuddin University, Makassar, Indonesia, unhas.ac.id

**Keywords:** balloon kyphoplasty, bridging ossification, endplate injury, osteoporosis, vertebral

## Abstract

**Introduction:**

Intervertebral bridging ossification (IBO) occasionally occurs after balloon kyphoplasty (BKP) for osteoporotic vertebral fractures (OVFs), contributing to stabilization. However, the predisposing factors remain unclear. This study aimed to identify preoperative factors associated with IBO formation.

**Methods:**

This was a retrospective cohort study of patients who underwent BKP for OVFs. Radiological evaluations included the location of the fractured vertebra, number of preexisting vertebral fractures, endplate damage, intervertebral disc injury, presence of diffuse idiopathic skeletal hyperostosis, lateral wedge angle and regional kyphosis angle, and were assessed using radiography, computed tomography and magnetic resonance imaging (MRI). Clinical outcomes were evaluated via the visual analogue scale (VAS) and Oswestry Disability Index (ODI) preoperatively and at 1 month and 1 year postoperatively.

**Results:**

Patients with IBO showed higher rates of thoracolumbar junction fractures (T11–L1) (*p* < 0.001), more preexisting vertebral fractures (*p* < 0.001), proximal endplate injury (*p* < 0.001), increased T2‐weighted signal intensity of the adjacent intervertebral disc (*p* = 0.015) and larger lateral wedge angles in supine (*p* = 0.008) and sitting positions (*p* = 0.001). At 1 month, VAS scores were higher in the IBO group (4.2 ± 1.8 vs. 2.6 ± 2.0, *p* = 0.001). Multiple regression analysis revealed preexisting vertebral fractures (*p* < 0.001) and proximal endplate injury (*p* = 0.002) as independent predictors of IBO formation. VAS scores at 1 month postoperatively were worse in the IBO group (*p* = 0.001), but no significant differences were observed at 1 year.

**Conclusion:**

Preexisting vertebral fractures and proximal endplate injury are key predictors of IBO formation after BKP. Although associated with higher short‐term pain, IBO appears to contribute to long‐term stabilization and pain relief, providing insights into postoperative outcomes and treatment strategies.

## 1. Introduction

Osteoporotic vertebral fractures (OVFs) represent a significant health challenge correlated with pain, augmented morbidity and disability [1]. As the population ages, the incidence of OVF progressively escalates, detrimentally affecting the daily functions of older people and amplifying their socioeconomic burden [1, 2]. Balloon kyphoplasty (BKP) is a minimally invasive surgical approach for patients with OVF who are severely affected by pain or demonstrate suboptimal responses to conservative management [3]. Multiple clinical studies have reported promising therapeutic outcomes for this procedure [4–7]. Although the precise mechanisms responsible for post‐BKP pain alleviation remain elusive, the prevailing consensus suggests a role in the stabilization of fractured vertebrae. However, because fractured vertebral instability after BKP can occasionally persist, knowledge of fracture site stabilization methods is important for determining the optimum surgical technique.

Occasionally, intervertebral bridging ossification (IBO) is observed after BKP, which potentially contributes to the stabilization of vertebral fractures [8, 9]. Previous case reports have described IBO after percutaneous vertebroplasty or percutaneous kyphoplasty for OVF [8, 10, 11]. In their report, characteristics and susceptibility factors were speculated. However, owing to an insufficient number of cases, the factors associated with IBO remain unclear. Therefore, we hypothesized that preoperative radiological characteristics of the fractured vertebral segment would be associated with the subsequent development of IBO after BKP for OVFs. The purpose of this study was to statistically evaluate the patient characteristics, radiological data and clinical outcomes of patients undergoing BKP for OVF and to identify factors associated with subsequent IBO.

## 2. Materials and Methods

### 2.1. Patients

Consecutive patients who underwent BKP for OVF between May 2011 and December 2017 at the two institutions were analysed.

Inclusion criteria were as follows:

1. Age ≥ 65 years;
2. Acute OVF within 1 month of symptom onset;
3. Availability of preoperative magnetic resonance imaging (MRI); and
4. Availability of at least 1 year of follow‐up.

Exclusion criteria were as follows:

1. Neurological deficit;
2. Pathological fracture;
3. Suspected underlying malignant disease;
4. Adjacent vertebral fracture on preoperative imaging;
5. Revision surgery after BKP; and
6. Postoperative teriparatide treatment.

Patients who received postoperative teriparatide were excluded because this anabolic agent stimulates new bone formation and could directly influence IBO formation, thereby confounding the association between baseline radiological factors and subsequent IBO. An incident vertebral fracture was diagnosed based on acute low back pain (LBP), a decrease of at least 20% or 4 mm in any vertebral height (anterior, central or posterior) on radiography and abnormal intensity within the vertebral bodies on MRI. The indication for BKP was persistent with LBP ≥ 4 on the visual analogue scale (VAS, 0–10), the presence of fractured vertebral instability and resistance to conservative treatments. BKP was performed via a percutaneous, bilateral transpedicular approach using introducer instruments, inflatable bone tamps, polymethylmethacrylate bone cement and delivery devices (Kyphon Balloon Kyphoplasty, Medtronic Spine LLC, Sunnyvale, CA, USA) [12]. All procedures were performed under general anaesthesia. All the participants received analgesics and tailor‐made elastic braces.

The study was conducted in accordance with the 1964 Declaration of Helsinki and its subsequent amendments or equivalent ethical standards. This study protocol was approved by the ethical committee for research of our institution, and written informed consent was obtained from all the patients.

### 2.2. Assessments

At enrolment, all patients underwent plain radiography, computed tomography (CT) and MRI of the spine. Patient‐reported outcomes were assessed using a VAS for LBP and the Oswestry Disability Index (ODI) [13, 14]. At 1 month and 1 year postoperatively, plain radiography and CT were repeated, and the VAS and ODI were reassessed. Postoperative adverse events, including adjacent vertebral fractures and symptomatic cement–related complications, were recorded during the 1‐year follow‐up.

Baseline data included age, sex, time from symptom onset to surgery, preoperative use of osteoporosis medication and bone mineral density (BMD). Lumbar spine and left proximal femur BMD were measured by dual‐energy X‐ray absorptiometry. Lumbar BMD was calculated as the mean value of L2–L4, excluding the fractured vertebra.

**FIGURE 1:**
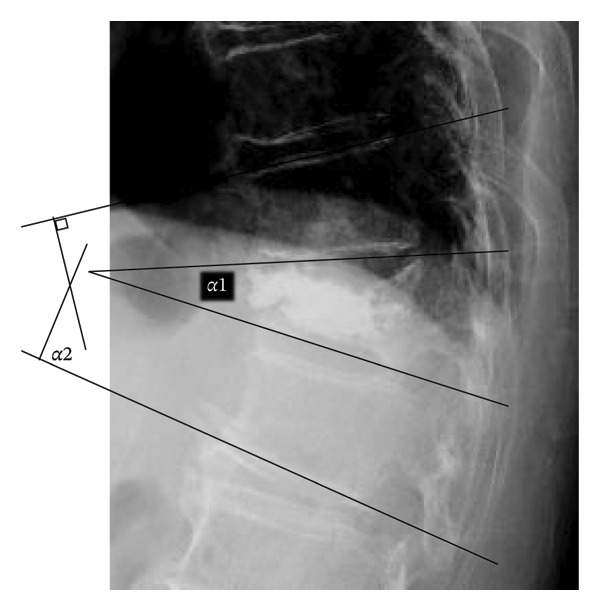
Parameters for sagittal alignment include the lateral wedge angle (α1) and the Cobb angle between proximal and distal adjacent vertebrae (α2: regional kyphosis angle) on lateral radiographs. These angular parameters were used to assess local sagittal alignment and postural instability at the fractured segment.

Sagittal alignment was assessed on lateral radiographs obtained in the supine and sitting positions. We measured the lateral wedge angle (*α*1; Figure 1) and the Cobb angle between the proximal and distal adjacent vertebrae (regional kyphosis angle, *α*2; Figure 1). We also calculated the differences in these angles between the two positions. CT images were used to evaluate the presence of IBO, the fractured spinal level, posterior wall injury, prevalent vertebral fractures, endplate injury and diffuse idiopathic skeletal hyperostosis (DISH) [15]. DISH was defined as continuous ossification bridging four or more vertebrae with preserved disc height and without osteosclerosis or bony fusion at the sacroiliac joints [16]. IBO was defined as complete ossified bridging of the intervertebral space, confirmed on sagittal or coronal CT images (Figure 2).

FIGURE 2Definition of intervertebral bridging ossification (IBO). IBO was defined as the complete bridging of the intervertebral space by ossification (arrowheads), confirmed on either sagittal (a) or coronal (b) CT images. The presence or absence of IBO at the fractured segment on follow‐up CT served as the primary radiological outcome for classifying patients into the IBO and non‐IBO groups.

**Figure:**
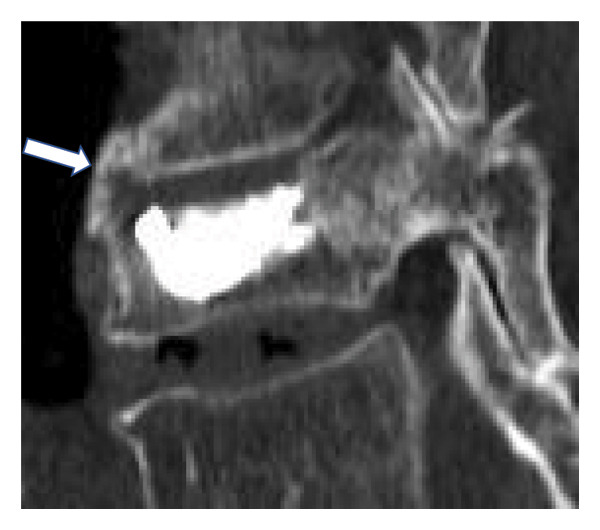
(a)

**Figure:**
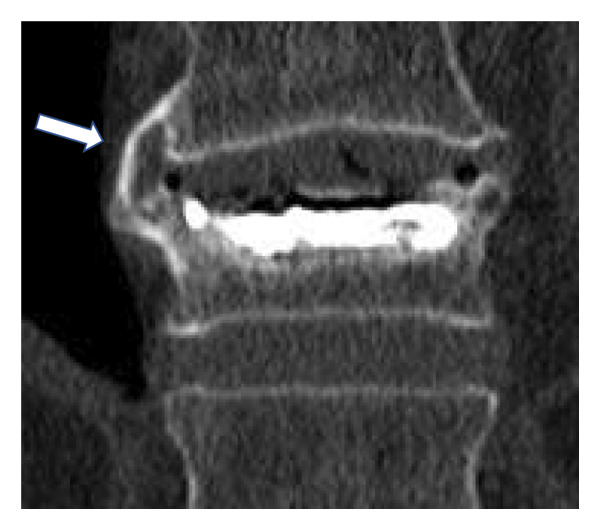
(b)

**FIGURE 3:**
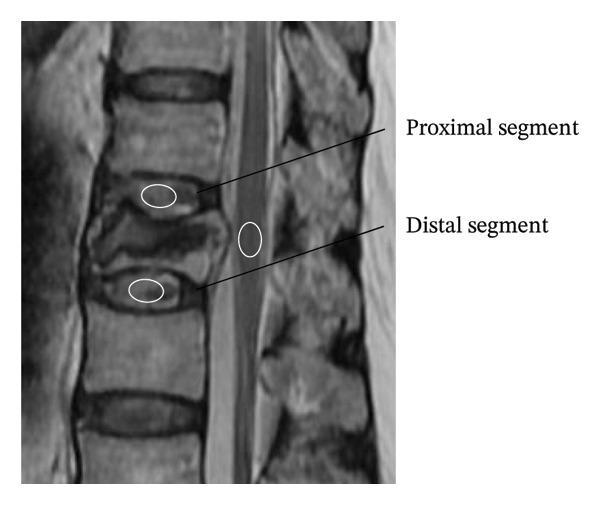
Area where the T2‐weighted signal intensity (SI) values were measured. Midsagittal T2‐weighted images were used for evaluation. The SI values were acquired by placing regions of interest in the center of the disc adjacent to the fractured vertebrae and the conus medullaris of the spinal cord, under standard conditions. The ratio of disc SI to cord SI (SIdisc/SIcord) was calculated and used as a quantitative indicator of disc changes adjacent to the fractured vertebra in the analysis of factors associated with IBO.

Intervertebral disc changes were evaluated using the T2‐weighted signal ratio on MRI. Midsagittal T2‐weighted images were used for the evaluation. Signal intensity (SI) values were acquired using the region of interest as the centre of the disc and the conus medullaris of the spinal cord under common conditions, as previously described [17]. We recorded disc SI at the proximal and distal intervertebral discs adjacent to the fractured vertebrae (Figure 3). The signal ratios were calculated using the following formulas [18, 19]: Signal ratio = SIdisc/SIcord.

Plain radiography, CT and MRI findings were evaluated by two experienced spine surgeons (T.M. and N.K.). The measurements of signal ratios, lateral wedge angles and regional kyphosis angles were performed independently, and the average of the two measurements was used for the evaluation. The presence of IBO, fractured spine level, posterior wall injury, prevalent spine fracture, endplate injury and comorbidity of DISH were determined by consensus.

### 2.3. Statistical Analysis

Continuous values were expressed as means and standard deviations. All data were analysed using SPSS Version 22.0 (IBM Corporation, Armonk, NY, USA) and JMP 17 (SAS Institute Inc., Cary, NC, USA). The inter‐rater reliability of signal ratios and angles was assessed using the intraclass correlation coefficient (2, 1). An intraclass correlation coefficient of < 0.50 was defined as low reliability, 0.50–0.75 as moderate reliability, 0.75–0.89 as good reliability and ≥ 0.90 as excellent reliability [20]. Between‐group comparisons of continuous and nominal variables were performed using the Wilcoxon rank‐sum test and Pearson’s chi‐squared test, respectively. For all experiments, a *p* value < 0.05 was considered statistically significant. To identify factors significantly associated with IBO, multivariate statistical analysis was performed on the factors that differed significantly between the groups with and without IBO.

## 3. Results

**FIGURE 4:**
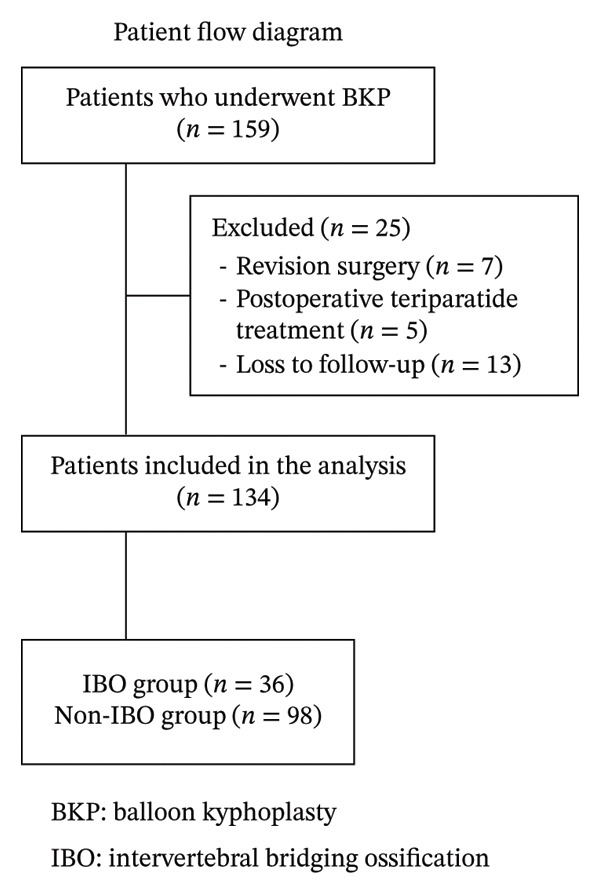
Patient flow diagram. A total of 159 patients underwent balloon kyphoplasty (BKP). Of these, 25 patients were excluded (revision surgery, *n* = 7; postoperative teriparatide treatment, *n* = 5; loss to follow‐up, *n* = 13), leaving 134 patients for analysis. Patients were classified into the intervertebral bridging ossification (IBO) group (*n* = 36) and the non‐IBO group (n = 98).

A total of 159 patients who underwent BKP were enrolled in this study. Of these, seven patients required revision surgery post‐BKP, five patients received postoperative teriparatide treatment, and 13 patients were excluded due to loss of follow‐up. Consequently, 134 patients successfully completed the one‐year follow‐up period (Figure 4). Among the 134 patients analysed in this study, the overall rate of IBO was 26.9%. Patients were divided into groups with and without IBO, and the two groups were compared. The baseline patient characteristics are shown in Table 1. There were no significant differences between the two groups in terms of age, sex, time from onset to surgery, preoperative use of osteoporosis medications, and BMDs, indicating that the two groups were comparable with respect to these general clinical characteristics.

**TABLE 1:** Baseline characteristics for the intervertebral bridging ossification (IBO) and non‐IBO groups.

	**IBO**	**Non-IBO**	**p** **value**

Number of patients	36 (26.9%)	98 (73.1%)	
Age (year)	81.6 ± 5.7	79.2 ± 6.4	0.094
Sex (female)	30 (83.3%)	68 (69.4%)	0.107
Time from onset to surgery (days)	36.4 ± 12.6	31.8 ± 9.6	0.075
Preoperative osteoporosis medication	12 (33.3%)	34 (34.7%)	0.883
BMD (g/cm^2^)			
Lumbar spine	0.9 ± 0.2	0.9 ± 0.2	0.927
Femur	0.7 ± 0.2	0.7 ± 0.2	0.871

Abbreviation: BMD, bone mineral density.

^∗^ *p* < 0.05.

The inter‐rater reliabilities for signal ratios, lateral wedge angles and regional kyphosis angles were good or excellent, confirming that these radiological measurements were reproducible and suitable for further analysis (Table 2). Radiological findings included whether the fractured vertebra was at the thoracolumbar junction (T11–L1), presence of posterior wall injury, the number of preexisting vertebral fractures, presence of proximal and distal endplate injuries of the fractured vertebra or DISH, signal ratio of the intervertebral disc adjacent to the fractured vertebra, lateral wedge angle and regional kyphosis angle in the supine and sitting positions and their differences (Table 3). Compared with the group without IBO (non‐IBO group), the group with IBO (IBO group) had a significantly higher incidence of fractures at the thoracolumbar junction (*p* < 0.001). There was no significant difference in the percentage of patients with posterior wall injuries between the two groups. The number of preexisting vertebral fractures was significantly higher in the bridge group (*p* < 0.001). However, when excluding patients without preexisting vertebral fractures and comparing only those with preexisting vertebral fractures, there was no significant difference in the number of preexisting vertebral fractures between the two groups. Fractures of the proximal vertebral endplate were significantly more common in the IBO group; however, there was no significant difference in the occurrence of distal vertebral endplate fractures between the two groups. In the signal ratio evaluation, the signal ratio of the proximal intervertebral disc adjacent to the fractured vertebra was significantly higher in the IBO group. The lateral wedge angle in the IBO group was significantly greater in both the supine and sitting positions. However, there was no significant difference between the two groups in the angle difference between the supine and sitting positions. In addition, there were no significant differences between the two groups in the regional kyphosis angle. As an additional analysis, we evaluated the incidence of adjacent vertebral fractures within 1 year. Adjacent vertebral fractures occurred in 34 patients (25.4%, 34/134). The incidence was significantly higher in patients with thoracolumbar junction fractures than in those with fractures at other levels (32.5% [26/80] vs. 14.8% [8/54], *p* = 0.037). Furthermore, the 1‐year incidence was significantly higher in the IBO group than in the non‐IBO group (Table 3). No symptomatic cement–related complications were observed during the follow‐up period. Analysis of the association between disc injury and signal ratio showed that patients with endplate injury on the proximal side of the fractured vertebral body had a significantly higher signal ratio for the proximally adjacent disc, and patients with endplate injury on the distal side had a significantly higher signal ratio for the distally adjacent disc (Table 4). Overall, these radiological findings suggest that fractures located at the thoracolumbar junction, the presence of preexisting vertebral fractures, and proximal endplate and adjacent disc injury are each associated with the development of IBO after BKP.

**TABLE 2:** Intraclass correlation coefficients (ICCs) with 95% confidence intervals (CIs) for signal ratios, lateral wedge angles and regional kyphosis angles.

	**ICC**	**95% CI**	**p** **value**

Signal ratio			
Proximal disc	0.821	0.756–0.870	<0.001^∗^
Distal disc	0.864	0.811–0.902	<0.001^∗^
Lateral wedge angle (degree)			
Supine position	0.972	0.960–0.980	<0.001^∗^
Sitting position	0.950	0.929–0.965	<0.001^∗^
Regional kyphosis angle (degree)			
Supine position	0.963	0.947–0.975	<0.001^∗^
Sitting position	0.961	0.944–0.973	<0.001^∗^

*Note:* Statistically significant *p* values (*p* < 0.05) are presented in bold, in response to the reviewer′s request to highlight significant results in the tables.

^∗^ *p* < 0.05.

**TABLE 3:** Comparison of radiological data between the intervertebral bridging ossification (IBO) and non‐IBO groups.

	**IBO**	**Non-IBO**	**p** **value**

Thoracolumbar junction (T11–L1)	30 (83.3%)	50 (51.0%)	<0.001^∗^
Posterior wall injury	12 (33.3%)	20 (20.4%)	0.120
Adjacent vertebral fractures	14 (38.9%)	20 (20.4%)	0.029^∗^
Number of preexisting fractures			
Total	1.3 ± 1.3	0.5 ± 0.9	<0.001^∗^
Excluding no fractures	2.1 ± 1.0	1.7 ± 0.9	0.106
Endplate injury			
Proximal	32 (88.9%)	53 (54.1%)	<0.001^∗^
Distal	9 (25.0%)	36 (36.8%)	0.202
DISH	4 (11.1%)	14 (14.3%)	0.633
Signal ratio			
Proximal disc	1.1 ± 0.3	1.0 ± 0.3	0.015^∗^
Distal disc	0.9 ± 0.3	0.9 ± 0.3	0.831
Lateral wedge angle (degree)			
Supine position	12.8 ± 8.1	8.5 ± 6.6	0.008^∗^
Sitting position	21.3 ± 6.4	16.0 ± 8.3	0.001^∗^
Difference	8.5 ± 4.2	7.4 ± 4.5	0.969
Regional kyphosis angle (degree)			
Supine position	16.2 ± 8.7	12.6 ± 9.0	0.102
Sitting position	29.0 ± 11.6	24.8 ± 11.6	0.158
Difference	12.6 ± 4.5	12.6 ± 6.9	0.650

*Note:* Statistically significant *p* values (*p* < 0.05) are presented in bold, in response to the reviewer′s request to highlight significant results in the tables.

Abbreviation: DISH, diffuse idiopathic skeletal hyperostosis.

^∗^ *p* < 0.05.

**TABLE 4:** Comparison of disc signal ratio between patients with and without endplate injuries.

**Signal ratio**	**Proximal endplate injury**		**p** **value**
	**With**	**Without**	
Proximal disc	1.1 ± 0.3	0.8 ± 0.2	<0.001^∗^
Distal disc	0.9 ± 0.2	0.9 ± 0.2	0.207

	**Distal endplate injury**		
	**With**	**Without**	

Proximal disc	0.9 ± 0.3	1.0 ± 0.2	0.090
Distal disc	1.0 ± 0.2	0.8 ± 0.3	<0.001^∗^

*Note:* Statistically significant *p* values (*p* < 0.05) are presented in bold, in response to the reviewer′s request to highlight significant results in the tables.

^∗^ *p* < 0.05.

The VAS and ODI were evaluated as clinical outcomes preoperatively and 1 month and 1 year postoperatively. Between‐group differences in VAS and ODI at each time point were tested as described in Section 2.3, and the corresponding *p*‐values are provided in Table 5. The VAS score 1 month after surgery was significantly worse in the bridge group, but the other parameters were not significantly different between the two groups. These clinical results indicate that patients who develop IBO tend to experience more persistent pain in the early postoperative period, although their longer‐term pain and disability outcomes are ultimately similar to those of patients without IBO.

**TABLE 5:** Comparison of clinical outcomes between the intervertebral bridging ossification (IBO) and non‐IBO groups.

	**IBO**	**Non-IBO**	**p** **value**

VAS (0–10)			
Before surgery	8.5 ± 1.1	8.1 ± 1.7	0.356
One month after surgery	4.2 ± 1.8	2.6 ± 2.0	0.001^∗^
One year after surgery	3.2 ± 1.3	3.5 ± 2.8	0.753
ODI (%)			
Before surgery	66.5 ± 18.3	64.3 ± 17.1	0.528
One month after surgery	40.1 ± 16.1	30.0 ± 15.2	0.058
One year after surgery	37.4 ± 9.5	31.6 ± 20.4	0.226

*Note:* Statistically significant p values (*p* < 0.05) are presented in bold, in response to the reviewer′s request to highlight significant results in the tables.

Abbreviations: ODI, Oswestry Disability Index; VAS, visual analogue scale.

^∗^ *p* < 0.05.

Multiple logistic regression analysis was performed to identify the factors involved in IBO formation. Multivariable logistic regression results are presented as odds ratios with 95% confidence intervals and *p* values (Table 6). The number of preexisting fractures and proximal endplate injuries were significantly associated with IBO formation, with odds ratios of 3.0 and 16.0, respectively. Thus, the presence of preexisting vertebral fractures and proximal endplate injury emerged as key independent predictors of IBO formation after BKP.

**TABLE 6:** Multiple regression analysis of factors related to bridging ossification.

Variables	Odds ratio	95% CI		*p* value
		Lower	Upper	
Thoracolumbar junction	1.8	0.5	6.2	0.347
Number of preexisting fractures	3.0	1.8	5.2	<0.001^∗^
Proximal endplate injury	16.0	2.8	91.0	0.002^∗^
Signal ratio in proximal disc	1.5	0.2	14.2	0.732
Lateral wedge angle in supine	1.1	0.9	1.2	0.452
Lateral wedge angle in sitting	1.0	0.9	1.2	0.508

*Note:* Statistically significant *p* values (*p* < 0.05) are presented in bold, in response to the reviewer′s request to highlight significant results in the tables.

^∗^ *p* < 0.05.

## 4. Discussion

In this study, we investigated the factors contributing to the formation of IBO following BKP for OVFs. The findings revealed that preexisting vertebral fractures and proximal endplate injuries were the most significant predictors of IBO formation. These results underscore the importance of biomechanical and pathological factors in the development of IBO, which may influence postoperative outcomes. Our analysis also highlights the complex interplay between endplate damage, intervertebral disc changes and subsequent ossification processes, providing new insights into the mechanisms underlying spinal stabilization after BKP.

A comparison of radiological factors with and without IBO showed significant differences in the number of preexisting vertebral fractures, presence of proximal endplate injury of the fractured vertebra, signal ratio of the disc proximally adjacent to the fractured vertebra and lateral wedge angle in the supine and sitting positions. There were significant differences in the number of previous vertebral fractures, but not when patients without previous vertebral fractures were excluded. These findings indicate that the presence of any preexisting vertebral fracture is associated with a higher likelihood of IBO, whereas a clear quantitative relationship with the number of fractures could not be demonstrated in this cohort. Clinically, this suggests that patients with a history of OVF should be regarded as being at increased risk of IBO after BKP, even if they have only a single prior fracture. Patients with IBO had a significantly higher incidence of vertebral fractures at the thoracolumbar junction. The thoracolumbar junction represents a biomechanical transition zone between the rigid thoracic spine and the spinal region with the greatest dynamic load [21]. Vertebral fractures at the thoracolumbar junction cause increased strain, leading to mechanical instability. Our study also revealed that preexisting vertebral fractures were significantly associated with IBO. Preexisting vertebral fractures are associated with sagittal malalignment and lead to an increased axial load on new vertebral fractures [22]. A mechanical axial load stimulates callus formation and new bone deposition during bone fracture healing [23]. These factors can stimulate IBO development, similar to bone fracture healing. The lateral wedge angle was significantly greater in patients with IBO in both supine and seated positions. This may also be due to differences in the mechanical properties of the IBO formation. An additional finding of this study was that adjacent vertebral fractures within 1 year occurred more frequently in the IBO group than in the non‐IBO group. This association may reflect that IBO formation was more common in patients with preexisting vertebral fractures and thoracolumbar junction fractures, which may indicate greater baseline fragility and mechanical stress across the motion segment. Alternatively, IBO formation may represent a response to greater segmental instability after OVF, which could also predispose adjacent levels to subsequent fracture. Given that this study was not designed to establish causality, this association should be interpreted cautiously.

Patients with IBO had a significantly higher percentage of endplate injuries on the proximal side of the fractured vertebra and a significantly higher signal ratio in the intervertebral disc proximally adjacent to the fractured vertebra. In addition, the signal ratio of the disc proximally adjacent to the fractured vertebra was significantly higher in patients with a proximal endplate injury to the fractured vertebra. These findings indicate an association between IBO, endplate injury and intervertebral disc signal ratio. An increased disc signal ratio may indicate disc injury. The literature reports a high incidence of endplate and disc injuries associated with OVFs [24, 25]. Cartilage endplates serve as a semipermeable interface that regulates nutritional transport from the vertebral body to the disc tissues [26]. In endplate injury, blood leakage from the vertebral body can form a haematoma, and an inflammatory reaction is initiated at the injury site. In addition, it has been demonstrated that mechanical damage to the intervertebral disc directly induces a localized inflammatory response, leading to elevated levels of pro‐inflammatory cytokines, such as interleukin‐1*β* (IL‐1*β*), IL‐6 and tumour necrosis factor‐*α* [27]. Inflammation causes the secretion of various growth factors and cytokines, which can affect the recruitment and differentiation of mesenchymal stem cells to the site [28]. The process will promote the formation of IBO, which is similar to the fracture healing mechanism [28, 29]. In the clinical setting of OVFs, disc signal alterations on MRI are considered to occur mainly when the vertebral endplate–disc complex is disrupted, suggesting that disc injury is more directly related to endplate damage than to the presence of vertebral fractures themselves. In this context, preexisting vertebral fractures may reflect a globally weakened spinal segment and severe osteoporosis, whereas proximal endplate injury represents local structural failure that can transmit mechanical and biological stress to the adjacent disc. The combination of a vulnerable background and endplate‐related disc injury may lead to intradiscal inflammation and a cytokine‐ and growth factor–rich microenvironment, which could facilitate IBO after BKP. Although histologic evaluation was not performed in this study, histologic examinations of osteoporotic fractured vertebral bodies have shown focal areas of endochondral new bone formation with neovascularization adjacent to necrotic bone, indicating that these lesions represent an active fracture healing process rather than simple dystrophic calcification [30]. In this context, IBO after BKP in our cohort is likely to reflect similar biologically active new bone formation across the injured motion segment. In previous studies, disc injuries associated with OVFs were classified using MRI according to Sander et al.’s report [31]. However, we evaluated MRI by quantitative values according to Kamei et al.’s report [17] because it can be difficult to find disc changes to determine the classification, especially in older patients due to disc degeneration. A high disc intensity indicates oedema and intradiscal bleeding [31]. Quantitative evaluation methods, such as T2/T1ρ mapping, may be useful in assessing the grade of disc injury. However, these methods require MRI scanning under specific conditions, and MRI images obtained under general conditions cannot be utilized. Therefore, in this study, signal ratios that can be evaluated using MRI images obtained under general conditions were employed [17, 32]. Although disc injury may be associated with IBO formation, multiple regression analysis showed a significant association between endplate injury and IBO but no significant association between disc signal ratio and IBO. Therefore, endplate injury may be more important than the disc signal ratio in predicting IBO formation. The relatively high percentage of patients with IBO in this study (26.9%) may be attributed to the large number of patients with endplate injuries and the detailed identification of IBO using CT.

In the evaluation of clinical outcomes, significant differences were found only in the VAS scores at 1 month postoperatively, with significantly greater VAS scores in patients with IBO. However, at 1 year postoperatively, there was no significant difference in the VAS or ODI between the two groups. As BKP often stabilizes the fracture site and reduces pain without IBO formation, it is reasonable that there was no significant difference in the clinical outcomes between the two groups at 1 year postoperatively. However, the fact that patients with IBO had a significantly higher VAS score at 1 month postoperatively, but at 1 year postoperatively, the VAS score had decreased to the same level as that in patients without IBO suggests that in patients with IBO, BKP alone was not sufficient to stabilize the fracture, leading to pain, but subsequent IBO formation might provide local stabilization and pain relief.

BKP for OVF is a treatment that provides early postoperative pain relief [33, 34]. However, if patients do not demonstrate adequate response to BKP, posterior fixation is typically added [35]. In this study, patients with IBO had a significantly higher VAS score at 1 month after surgery compared to those without IBO. This suggests that patients with IBO may benefit from additional posterior fixation. However, it is worth noting that the subsequent formation of the IBO may reduce pain by stabilizing the fracture site. Therefore, the results of this study indicate that continued conservative treatment, such as bracing, could be considered as an option for patients with a predisposition to IBO formation, even if they do not experience sufficient pain relief after BKP.

Previous studies have shown that osteoporotic vertebral deformity with radiographic endplate fracture identifies vertebrae at increased risk of subsequent deformity progression and new vertebral fractures, highlighting the importance of careful assessment of baseline endplate integrity [36]. In this context, our finding that proximal endplate injury predicts IBO after BKP supports the concept that preexisting endplate damage reflects a mechanically vulnerable segment, whereas prevention of new endplate fractures primarily depends on optimized anti‐osteoporotic therapy and appropriate surgical technique [37].

From a practical standpoint, our findings suggest that patients with preexisting vertebral fractures and proximal endplate injury are more likely to exhibit a delayed stabilization pattern characterized by IBO after BKP. For such patients, clinicians may anticipate a higher likelihood of IBO formation, consider closer early postoperative radiographic follow‐up, and, when early pain relief is insufficient, temporarily reinforce conservative management (e.g. continued bracing). Awareness that IBO‐related stabilization can occur gradually over time may also help surgeons carefully weigh the need for immediate additional posterior fixation and provide patients with more accurate counselling regarding the possibility of early persistent pain followed by later improvement.

This study had some limitations. First, this was a retrospective observational study in which the patients had variations in obesity, pharmaceutical agent use and timing of MRI examination and BKP, with potential selection bias. Second, the sample size was relatively small, and only 36 patients developed IBO. Therefore, further studies with larger cohorts and longer follow‐up periods are warranted to validate these results and to explore the potential of incorporating IBO‐related factors into clinical decision‐making. In addition, CT assessments were performed through a consensus review process, and because independent ratings were not available, interobserver agreement statistics could not be calculated. Radiographic assessment of cement leakage (including asymptomatic leakage or cement embolism) was not routinely performed; although no symptomatic cement–related complications were observed during follow‐up, asymptomatic events may have been missed. Finally, while disability and activities of daily living were assessed using the ODI at baseline and at 1 month and 1 year postoperatively, objective functional tests at 1 year were not performed.

In conclusion, this study identified preexisting vertebral fractures and proximal endplate injuries as key predictors of IBO after BKP for OVFs. IBO formation, while associated with persistent pain at 1 month postoperatively, appears to contribute to long‐term stabilization and pain relief. These findings offer valuable insights for predicting outcomes and tailoring treatment strategies for OVF patients. Future prospective, multicentre studies are needed to clarify how these IBO‐related factors can be incorporated into clinical decision‐making, including patient selection and postoperative management.

## Author Contributions

Toshiaki Maruyama: conceptualization, data curation, formal analysis, investigation, methodology and writing–original draft. Naosuke Kamei: conceptualization, formal analysis, methodology and writing–original draft. Toshio Nakamae: data curation, investigation and writing–review and editing. Yoshinori Fujimoto: investigation, writing–review and editing and supervision. Kiyotaka Yamada: methodology and writing–review and editing. Kazuto Nakao: Investigation and writing–review and editing. Fadlyansyah Farid: investigation and writing–review and editing. Hiroki Fukui: investigation and writing–review and editing. Nobuo Adachi: investigation, writing–review and editing and supervision.

## Funding

This research did not receive any specific grant from funding agencies in the public, commercial or not‐for‐profit sectors.

## Ethics Statement

This study was approved by the Ethical Committee for Research of Hiroshima University (approval number: E2022‐0058).

## Conflicts of Interest

The authors declare no conflicts of interest.

## Supporting Information

STROBE Statement—checklist of items that should be included in reports of observational studies.

## Supporting information


**Supporting Information** Additional supporting information can be found online in the Supporting Information section.

## Data Availability

The data that support the findings of this study are available upon request from the corresponding author. The data are not publicly available due to privacy or ethical restrictions.
