# Supplementary material for: Relationship of Preexisting Vertebral Fractures and Endplate Injury to Intervertebral Bridging Ossification After Balloon Kyphoplasty for Osteoporotic Vertebral Fractures
Source: J Osteoporos. 2026 Feb 23;2026:1886674. doi: 10.1155/joos/1886674 (PMC12927987; doi:10.1155/joos/1886674)
Supplement: Supplementary file 1 — Supporting Information Additional supporting information can be found online in the Supporting Information section. [file JOOS-2026-1886674-s001.pdf]

STROBE Statement—Checklist of items that should be included in reports of ***cohort studies***

	Item No	Recommendation	Page/Line
**Title and abstract**	1	(*a*) Indicate the study's design with a commonly used term in the title or the abstract	Manuscript: p. 3
		(*b*) Provide in the abstract an informative and balanced summary of what was done and what was found	Manuscript: p. 3
Background/rationale	2	Explain the scientific background and rationale for the investigation being reported	Manuscript: p. 3–4
Objectives	3	State specific objectives, including any prespecified hypotheses	Manuscript: p. 3–4
Study design	4	Present key elements of study design early in the paper	Manuscript: p. 4
Setting	5	Describe the setting, locations, and relevant dates, including periods of recruitment, exposure, follow-up, and data collection	Manuscript: p. 4–6
Participants	6	(*a*) Give the eligibility criteria, and the sources and methods of selection of participants. Describe methods of follow-up	Manuscript: p. 4–5
		(*b*) For matched studies, give matching criteria and number of exposed and unexposed	N/A (not matched)
Variables	7	Clearly define all outcomes, exposures, predictors, potential confounders, and effect modifiers. Give diagnostic criteria, if applicable	Manuscript: p. 4–7 Tables: p. 1–3
Data sources/ measurement	8*	For each variable of interest, give sources of data and details of methods of assessment (measurement). Describe comparability of assessment methods if there is more than one group	Manuscript: p. 6–7 Tables: p. 1–3
Bias	9	Describe any efforts to address potential sources of bias	Manuscript: p. 14–15
Study size	10	Explain how the study size was arrived at	Manuscript: p. 4, 7 (Consecutive patients; flow diagram)
Quantitative variables	11	Explain how quantitative variables were handled in the analyses. If applicable, describe which groupings were chosen and why	Manuscript: p. 7 (See Statistical analysis)
Statistical methods	12	(*a*) Describe all statistical methods, including those used to control for confounding	Manuscript: p. 7 (See Statistical analysis)
		(*b*) Describe any methods used to examine subgroups and interactions	Manuscript: p. 7–9 (See Results for subgroup analyses)
		(*c*) Explain how missing data were addressed	Manuscript: p. 7; Loss to follow-up described; missing data handling not separately reported
		(*d*) If applicable, explain how loss to follow-up was addressed	Manuscript: p. 7, 18 (Loss to follow-up;

			flow diagram)
		(*e*) Describe any sensitivity analyses	N/A (no sensitivity analyses)
Participants	13*	(a) Report numbers of individuals at each stage of study—eg numbers potentially eligible, examined for eligibility, confirmed eligible, included in the study, completing follow-up, and analysed	Manuscript: p. 7, 18
		(b) Give reasons for non-participation at each stage	Manuscript: p. 7, 18
		(c) Consider use of a flow diagram	Manuscript: p. 18
Descriptive data	14*	(a) Give characteristics of study participants (eg demographic, clinical, social) and information on exposures and potential confounders	Manuscript: p. 7–9<br>Tables: p. 1
		(b) Indicate number of participants with missing data for each variable of interest	Manuscript: p. Not reported<br>(Missing data per variable not provided)
		(c) Summarise follow-up time (eg, average and total amount)	Manuscript: p. 4, 7<br>(Follow-up stated as 1 year; mean follow-up not provided)
Outcome data	15*	Report numbers of outcome events or summary measures over time	Manuscript: p. 7–9<br>Tables: p. 2–3
Main results	16	(*a*) Give unadjusted estimates and, if applicable, confounder-adjusted estimates and their precision (eg, 95% confidence interval). Make clear which confounders were adjusted for and why they were included	Manuscript: p. 7–9<br>Tables: p. 3
		(*b*) Report category boundaries when continuous variables were categorized	Manuscript: p. 4–5<br>(Eligibility criteria and thresholds)
		(*c*) If relevant, consider translating estimates of relative risk into absolute risk for a meaningful time period	N/A
Other analyses	17	Report other analyses done—eg analyses of subgroups and interactions, and sensitivity analyses	Manuscript: p. 8–9<br>Tables: p. 2–3
Key results	18	Summarise key results with reference to study objectives	Manuscript: p. 10–15
Limitations	19	Discuss limitations of the study, taking into account sources of potential bias or imprecision. Discuss both direction and magnitude of any potential bias	Manuscript: p. 14–15
Interpretation	20	Give a cautious overall interpretation of results considering objectives, limitations, multiplicity of analyses, results from similar studies, and other relevant evidence	Manuscript: p. 10–15
Generalisability	21	Discuss the generalisability (external validity) of the study results	Manuscript: p. 14–15<br>(External validity discussed briefly)

Funding	22	Give the source of funding and the role of the funders for the present study and, if applicable, for the original study on which the present article is based	Manuscript: p. 20 (Funding)

*Give information separately for exposed and unexposed groups.

Note: An Explanation and Elaboration article discusses each checklist item and gives methodological background and published examples of transparent reporting. The STROBE checklist is best used in conjunction with this article (freely available on the Web sites of PLoS Medicine at http://www.plosmedicine.org/, Annals of Internal Medicine at http://www.annals.org/, and Epidemiology at http://www.epidem.com/). Information on the STROBE Initiative is available at http://www.strobe-statement.org.
